# The Effect of a Commercially Available Bacteriophage and Bacteriocin on *Listeria monocytogenes* in Coleslaw

**DOI:** 10.3390/v11110977

**Published:** 2019-10-23

**Authors:** Rhea Lewis, Andrei Sorin Bolocan, Lorraine A. Draper, R. Paul Ross, Colin Hill

**Affiliations:** 1 APC Microbiome Ireland, University College Cork, T12 YT20 Cork, Ireland; rhea.lewis@umail.ucc.ie (R.L.); andrei.s.bolocan@gmail.com (A.S.B.); L.Draper@ucc.ie (L.A.D.); p.ross@ucc.ie (R.P.R.); 2 School of Microbiology, University College Cork, T12 YN60 Cork, Ireland

**Keywords:** phage, bacteriocin, *Listeria monocytogenes*, food safety

## Abstract

Changing consumer attitudes show an increased interest in non-chemical antimicrobials in food preservation and safety. This greater interest of consumers in more ‘natural’ or ‘clean-label’ food interventions is complicated by concurrent demands for minimally processed, ready-to-eat (RTE) foods with long shelf lives. Two viable interventions are bacteriophage (phage) and bacteriocins, a number of which have already been approved for use in food safety. Listeriosis is a serious foodborne infection which affects at-risk members of the population. Listeriosis incidence has increased between 2008 and 2015 and has a case fatality rate of up to 20% with antibiotic intervention. Here, we tested an intervention to attempt to control a pathogenic *Listeria monocytogenes* strain in a food model using two of these alternative antimicrobials. Phage P100 on its own had a significant effect on *L. monocytogenes* ScottA numbers in coleslaw over a 10-day period at 4 °C (*p* ≤ 0.001). A combination of P100 and Nisaplin^®^ (a commercial formulation of the lantibiotic bacteriocin, nisin) had a significant effect on the pathogen (*p* ≤ 0.001). P100 and Nisaplin^®^ in combination were more effective than Nisaplin^®^ alone, but not P100 alone.

## 1. Introduction

*Listeria monocytogenes* is a small, Gram positive rod-shaped bacteria and the causative agent of listeriosis [1,2]. Listeriosis is a serious foodborne illness which can occur as sporadic cases or as outbreaks, and most commonly affects the most susceptible members of the population; namely, neonates, the immunocompromised, the elderly, and pregnant women. The majority of listeriosis cases manifest one of three clinical syndromes: Maternofetal or neonatal listeriosis, blood stream infection, or meningoencephalitis. *Listeria* is of significant concern, despite the fact that fewer than 2300 cases were reported in the European Union (EU)/European Economic Area (EEA) per year between 2008 and 2015 [3]. This concern is due to an overall annual case fatality rate of 12–20%, the involvement of at risk groups, and an increase in confirmed cases between 2008 and 2015. *L. monocytogenes* has been associated with a wide range of foods such as meat, seafood, vegetables, egg products, dairy products, and ready-to-eat (RTE) foods [4,5]. Interest is growing in biopreservation: The use of the anti-microbial properties of micro-organisms and their metabolites for the preservation of food [6]. This is, in part, due to the growing popularity of ready-to-eat (RTE) and minimally processed foods, which often carry an increased risk of microbiological contamination and are usually not cooked before consumption. Another reason for the rising interest in biopreservation is the trend towards ‘clean label’ and more ‘natural’ foods because of rising consumer distrust in traditional, artificial additives and preservatives [7]. Bacteriophages (phages) are attracting interest for use in food preservation and food safety [8]. Phages are effective at killing bacteria, are specific to their host, and, as a result of their ubiquity in nature, are commonly encountered and ingested, making them attractive for use in food safety. The anti-*Listeria* phage Listex P100 contains a single phage, P100, and was granted Generally Regarded as Safe (GRAS) status by the American Food and Drug Agency (FDA) in 2006 [9]. Bacteriocins are another alternative to traditional additives and preservatives for food safety [10]. Bacteriocins are ribosomally synthesised antimicrobial peptides with a broad or narrow spectrum of action produced by bacteria. Many bacteriocins are produced by lactic acid bacteria used in food fermentations that have been granted Generally Regarded as Safe (GRAS) status by the FDA or have been granted Qualified Presumption of Safety (QPS) by The European Food Safety Authority (EFSA) [11]. Nisaplin^®^ containing nisin has been approved as a food preservative. Here, the activity of two commercially available ‘clean label’ antimicrobial compounds were investigated against *L. monocytogenes*, a foodborne pathogen of great importance and increasing incidence, in coleslaw. The objectives of this study were to (1) investigate the effect of P100 alone at a high MOI (multiplicity of infection) in coleslaw, (2) find a suitable combination of concentrations of P100 and Nisaplin^®^, and (3) investigate the efficacy of the combination in comparison to an untreated control, P100 alone and Nisaplin^®^ alone.

## 2. Materials and Methods

### 2.1. Bacteriophage Propagation and Bacteriophage Assays

Bacterial strains were grown in TSB (tryptic soy broth) and TSA (tryptic soy broth 1.5% agar *w*/*v*) at 37 °C. Plaque assays were carried out by overlaying 4 mL TSA (0.4% agar *w*/*v*) supplemented with Calcium boroglucinate (final concentration 10 mM). Plates were incubated at 30 °C for 24 h. P100 was isolated from Listex. P100 was propagated on *L. monocytogenes* strain 33116 ATCC 19117. To propagate P100, plaque assays were carried out. After 24 h incubation, 5 mL SM buffer (50 mM Tris-HCl; 100 mM NaCl; 8.5 mM MgSO_4_; pH 7.5) and 2% (*v*/*v*) chloroform were added to plates with confluent plaques and incubated at room temperature for 10 min. Phage suspension was removed from the plates, centrifuged at 4700× *g* for 10 min at 4 °C in a swing-bucket centrifuge, and filtered twice through a 0.45 µm pore diameter filter. Chloroform was removed using a VivaSpin^®^ 6 column.

### 2.2. Host Range of P100

The host range of P100 was established by carrying out plaque assays and comparing the phage titre on the strain to be tested to the phage titre on strain 33116 ATCC 19117 (Table 1) (Titre on test strain/Titre on strain 33116 ATCC 19117).

### 2.3. Coleslaw Food Trial with P100

Coleslaw was purchased from a supermarket. *L. monocytogenes* ScottA was grown overnight in TSB at 37 °C, centrifuged at 5500× *g* for 20 min, and the resulting pellet resuspended in PBS. Resuspended ScottA was then diluted to ~10^7^ CFU/mL in PBS and 100 µL added per 10 g of coleslaw. P100 was diluted to ~10^8^ PFU/mL in SM buffer. The control group was treated with 1 mL SM buffer per 10 g of coleslaw, and the test group was treated with 1 mL diluted P100 per 10 g of coleslaw. Bacteria and phage were added immediately after one another. PBS and SM buffer were added in place of ScottA and P100 as negative controls, which also served as a means for sterility monitoring throughout the experiment. Coleslaw was manually mixed for 1 min after these additions; 10 g samples of coleslaw were aliquoted and stored at 4 °C for 10 days. Then, 1 mL of liquid was pipetted off from the top of the coleslaw sample and used for spread plating of undiluted coleslaw to count low numbers of ScottA and diluting to count P100. To count P100, 500 µL of coleslaw liquid was added to 4.5 mL SM buffer, vortexed, centrifuged at 4700× *g* for 10 min at 4 °C in a swing-bucket centrifuge and filtered through a 0.45 µm pore diameter filter, and spot assays carried out against ScottA. To count ScottA, 90 mL PBS was added to 10 g of coleslaw and stomached for 90 s, and then serial dilutions were prepared in PBS. Oxford agar and Oxford Listeria Selective Supplement were used to count ScottA by spread plating 100 µL of the relevant dilution. Plates were incubated at 37 °C for 24 h. To check for resistance, colonies were streaked on Oxford agar and Oxford Listeria Selective Supplement from Day 8 and Day 10 food trial count plates and spot assays carried out in triplicate against P100 lysates.

### 2.4. Rate of Resistance to P100

The rate that resistance to P100 occurs was assessed using the efficiency of lysogeny protocol of Dalmasso et al. [12]. Plates were incubated at 30 °C for 24 h. The percentage of bacteriophage insensitivity was calculated as follows: (CFU on phage seeded plates/CFU on phage-free control plates) × 100. Twenty colonies were picked from the phage seeded plate and spot assays carried out to evaluate the colonies’ sensitivity to P100.

### 2.5. P100 and Nisaplin^®^ Checkerboard Assay in Broth

P100 and Nisaplin^®^ checkerboard assay was performed in TSB as per Draper et al. in triplicate [13]. A 2-fold serial dilution of Nisaplin^®^ was made horizontally in sterile water (50 µL) in a 96-well microtitre plate. A 10-fold serial dilution of P100 was made vertically in SM buffer in a microtitre plate and 50 µL added vertically to the Nisaplin^®^ dilution plate. Bacteria were grown overnight, subcultured into TSB, and allowed to grow to an OD_600 nm_ of ~0.5. Bacteria were then diluted and 100 µL added to each microtitre well, resulting in a final concentration of 10^5^ CFU/mL. The final concentrations of Nisaplin^®^ were from 0 to 1600 µg/mL and P100 from MOI 0 to MOI 100, with the first well containing no antimicrobial agents. Wells containing media without bacteria were used to check for sterility. Growth was assessed visually after incubation for 24 h at 37 °C.

### 2.6. P100 and Nisaplin^®^ Checkerboard Assay in Coleslaw Liquid

Fresh coleslaw was centrifuged at 5400× *g* for 5 min at 4 °C in a swing-bucket centrifuge to separate solid and liquid parts. Then, 1 mL of coleslaw liquid was added to 24-well plates. ScottA was grown overnight in TSB at 37 °C, centrifuged at 5500× *g* for 20 min, and the resulting pellet resuspended in PBS. Resuspended ScottA was then diluted to ~10^7^ CFU/mL in PBS and 10 µL added per 1 mL of coleslaw. The starting concentration of ScottA in coleslaw liquid was ~10^5^ CFU/mL. A 1:10 serial dilution of P100 was made horizontally in SM buffer in a microtitre plate. In a second microtitre plate, Nisaplin^®^ dilutions were made in sterile water. Next, 10 µL of P100 and 20 µL of Nisaplin^®^ was added per 1 mL of coleslaw. The plate was stored at 4 °C for 24 h. After 24 h, 20 µL from each sample was serially diluted in 180 µL PBS in 96-well plates, and 10 µL of coleslaw dilutions were pipetted on Oxford agar and Oxford Listeria Selective Supplement and allowed to dry. Plates were incubated at 37 °C for 24 h. The combination of P100 and Nisaplin^®^ was evaluated using the Fractional Inhibitory Concentration (FIC) index [12]. The FIC looks at the interaction of antimicrobial compounds in the inhibition of a bacterial strain. It is defined by the equation: FIC = FICX + FICY = (X/MICX) + (Y/MICY). (MICx) is the minimum inhibitory concentration of the antimicrobial alone, while (X) is the lowest level of antimicrobial X in combination with another to achieve an inhibitory effect. FIC index results are interpreted as follows: FIC ≤ 0.5 is synergy, 0.5 < FIC ≤ 0.75 is partial synergy, 0.75 < FIC ≤ 1.0 is additive, FIC > 1.0 is indifferent, and FIC > 4 is antagonistic.

### 2.7. Coleslaw Food Trial with P100 and Nisaplin^®^ in Combination

P100 and Nisaplin^®^ combination food trial was carried out as per the P100 food trial with some adjustments. ScottA was diluted to ~10^7^ CFU/mL in PBS and 100 µL added per 10 g of coleslaw, as previously described. P100 was diluted to ~10^7^ PFU/mL in SM buffer. A 500 µg/mL stock solution of Nisaplin^®^ was prepared in sterile water, and 500 µL SM buffer and 500 µL sterile water per 10 g of coleslaw was added to the negative control samples. Then, 500 µL diluted P100 and 500 µL sterile water was added per 10 g of coleslaw to the P100 only samples; 500 µL SM buffer and 500 µL Nisaplin^®^ stock solution per 10 g of coleslaw was added to the Nisaplin^®^ only samples; and 500 µL diluted P100 and 500 µL Nisaplin^®^ stock solution per 10 g of coleslaw was added to the P100 and Nisaplin^®^ samples. Samples were stored at 4 °C for 10 days. To check for resistance, colonies were streaked on Oxford agar and Oxford Listeria Selective Supplement from Day 10 food trial count plates and spot assays carried out in triplicate against P100 lysates. To test for Nisaplin^®^ sensitivity spot assays were carried out using 10 µL of 50 µg/mL solution of Nisaplin^®^ on strain overlays similarly to phage spot assays.

### 2.8. Statistical Analysis

Bacterial and phage counts were determined by triplicate plating and all experiments were independently performed three times. Results are presented as mean values of these three experiments, and error bars in the figures indicate standard error of the mean (SEM). For CFU/g, graphs values were normalised before plotting. For coleslaw treated with P100 at MOI of 50, Student’s *t*-test (unpaired, two-tailed) was used to determine the significance of differences between controls and phage-treated samples. For coleslaw treated with P100 and Nisaplin^®^ in combination, one-way ANOVA was used to determine the significance of differences between controls and treated samples.

## 3. Results

### 3.1. Host Range of P100

P100 is suited to use in food safety due to its broad host range [14]. A range of strains of *Listeria* were tested for sensitivity to P100 (Table 1). These strains were originally isolated from a range of sources. P100, as expected, showed activity against many, but not all, pathogenic and non-pathogenic *Listeria* strains.

### 3.2. Coleslaw Food Trial with P100

**Figure 1:**
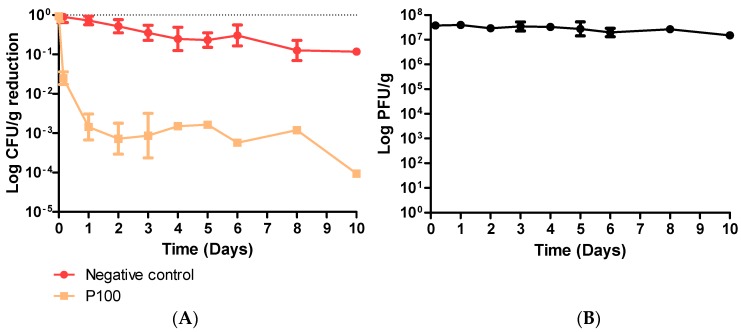
(**A**) Effect of P100 on ScottA reduction in coleslaw stored at 4 °C over a 10-day period. Error bars represent SEM. Coleslaw was spiked with bacteria (7.1 × 10^5^ CFU/g) and phage (4.0 × 10^7^ PFU/g). (**B**) P100 titre was also measured throughout the experiment.

Coleslaw was experimentally contaminated with ScottA at 7.1 × 10^5^ CFU/g. Over a 10-day period at 4 °C, ScottA was reduced approximately 10-fold to 7.6 × 10^4^ CFU/g in the untreated control (Figure 1A). The addition of phage P100 at an MOI of 50 significantly reduced ScottA in coleslaw stored under the same conditions (*p* ≤ 0.001). A significant reduction in ScottA was obvious within 2 h after phage addition (*p* = 0.0014) and numbers continued to fall for the first 48 h. Specifically, within 2 h of addition of P100, the ScottA numbers were reduced from 7.1 × 10^5^ to 2.0 × 10^4^ CFU/g (Table S1). Listeria numbers remained low throughout the test period. The reductions were statistically significant at each sampling day, with an overall reduction from 7.1 × 10^5^ CFU/g to an undetectable level (less than 30 colonies) after 10 days (Table S1). P100 titre was tested throughout the 10-day trial (Figure 1B). P100 titre remained high, at between 3.8 × 10^7^ PFU/g and 1.6 × 10^7^ PFU/g, during the 10-day trial. All 15 isolates were grown from individual colonies from Day 8 and all 20 colonies from Day 10, and were tested for resistance to P100 by spot assay (Table S2). Less than 30 colonies were present on Day 8 and Day 10, so were not counted and used for statistical analysis; however, they were picked and tested for resistance to P100. No isolates were resistant to P100 after 8 or 10 days of the trial. All colonies showed similar efficiency of plaquing of P100 as naïve sensitive ScottA, which had not been included in the food trial.

The rate that resistance to P100 occurs was assessed against ScottA. The bacterial counts on phage seeded plates and on phage-free control plates were 4.7 × 10^2^ CFU/mL and 4.0 × 10^8^ CFU/mL, respectively. This gave a resistance rate of 0.0001%. Twenty colonies from the phage seeded plate were tested for sensitivity to P100 by spot assay (Table S3). Eleven of the 20 colonies were resistant to P100 and formed no plaques or zones on spot assay. Six of the 20 colonies showed reduced sensitivity to P100 (efficiency of plaquing <0.4) compared to naïve ScottA. Three of the 20 colonies were more sensitive to P100 (efficiency of plaquing >1) than naïve ScottA.

### 3.3. P100 and Nisaplin^®^ Checkerboard Assays in Broth and Coleslaw

The minimum inhibitory concentration (MIC) of P100 and Nisaplin^®^ was investigated in TSB incubated at 30 °C for 24 h. The starting concentration of ScottA in broth and coleslaw was ~10^5^ CFU/mL. The MIC of P100 alone trended towards an MOI of 100. The MIC of Nisaplin^®^ alone trended towards 400 µg/mL. No synergistic effect was seen when P100 and Nisaplin^®^ were used in combination in checkerboard assays in broth.

To prepare for a food trial using P100 and Nisaplin^®^ in combination against ScottA in coleslaw, a checkerboard assay was carried out in coleslaw stored at 4 °C for 24 h (Table 2). Survival was measured by counting CFU/mL of each combination. The MIC for P100 occurred at an MOI of 10, while the MIC of Nisaplin^®^ alone was 50 µg/mL. The combination of P100 at an MOI of 1 and Nisaplin^®^ at a concentration of 25 µg/mL reduced the amount of each antimicrobial required to inhibit ScottA growth. The FIC of P100 at an MOI of 1 in combination with Nisaplin^®^ at a concentration of 25 µg/mL was 0.6, which represents partial synergy against ScottA. Based on this data, the concentrations of P100 at an MOI of 2.5 and 25 µg/mL Nisaplin^®^ were chosen for a food trial using P100 and Nisaplin^®^ in combination.

### 3.4. Coleslaw Food Trial with P100 and Nisaplin^®^ in Combination

**Figure 2:**
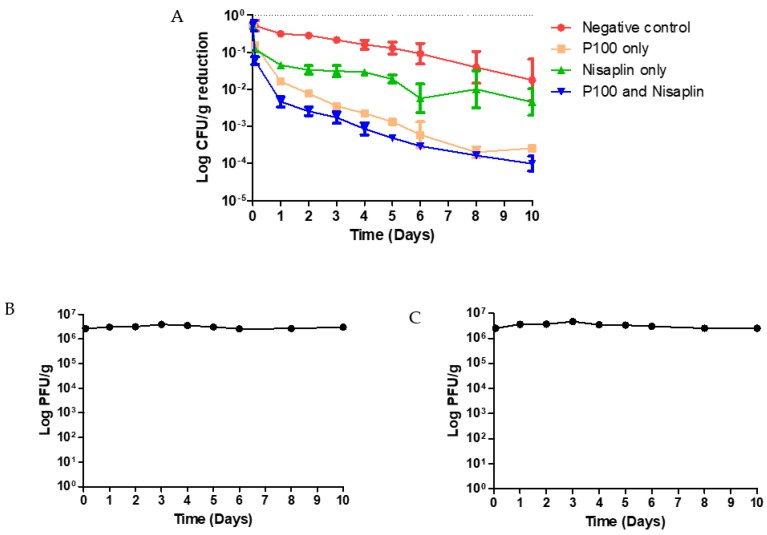
(**A**) Effect of P100 and Nisaplin^®^ in combination on ScottA reduction in coleslaw stored at 4 °C over a 10-day period. Coleslaw was spiked with bacteria (9.4 × 10^5^ CFU/g) and phage (~2.6 × 10^6^ PFU/g). Error bars represent SEM. P100 titre was also measured throughout the experiment of (**B**) P100 MOI 2.5 alone and (**C**) P100 MOI 2.5 and Nisaplin^®^ 25 µg/mL in combination.

Coleslaw was experimentally contaminated with ScottA at 9.4 × 10^5^ CFU/g. Over the 10-day period at 4 °C, ScottA in the untreated control was reduced to 7.4 × 10^4^ CFU/g, representing a reduction of more than 1 log (Figure 2A). The combination of P100 at an MOI of 2.5 and 25 µg/mL Nisaplin^®^ significantly reduced (*p* ≤ 0.001) the ScottA burden in coleslaw stored at 4 °C over a period of 10 days. Again, as seen with the P100 alone at an MOI of 50, a reduction in ScottA was visible 2 h after the antimicrobials were added, and reductions continued until the end of the 10-day period. Within 2 h of P100 and Nisaplin^®^ addition, ScottA numbers were reduced from 9.4 × 10^5^ to 1.0 × 10^5^ CFU/g (Table S4). ScottA numbers were reduced to 1.2 × 10^2^ CFU/g by day 10.

P100 alone also significantly reduced the ScottA numbers in coleslaw (*p* ≤ 0.001) over the 10-day period. This reduction was visible 2 h after P100 addition (9.4 × 10^5^ to 2.5 × 10^5^ CFU/g). There was no statistically significant difference in the levels of reduction in ScottA between P100 alone and P100 and Nisaplin^®^ in combination (*p* > 0.05).

Nisaplin^®^ alone did not significantly reduce ScottA numbers over the 10-day period (*p* > 0.05). However Nisaplin^®^ did significantly reduce ScottA numbers over the first 5 days (*p* < 0.01). The reduction was not significant on days 6, 8, 10, or overall (*p* > 0.05). ScottA numbers were reduced (9.4 × 10^5^ to 2.0 × 10^5^ CFU/g) within 2 h after Nisaplin^®^ addition.

Ten colonies from each test group on Day 10 were tested for resistance to P100 and Nisaplin^®^ by spot assay (Table S5). No colonies were found to be resistant to P100 after 10 days of the trial. All colonies showed similar efficiency of plaquing of P100 to naïve ScottA, which had not been included in the food trial. No colonies were found to be resistant to Nisaplin^®^ after 10 days of the trial. All colonies showed similar Nisaplin^®^ zones of inhibition to naïve ScottA, which had not been included in the food trial.

## 4. Discussion

The “Commission Regulation (EC) No 2073/2005 of 15 November 2005 on microbiological criteria for foodstuffs” outlines the acceptable levels in the EU for common pathogenic bacteria in a range of foods and at different points in their manufacturing and storage. The limit for *L. monocytogenes* is 100 CFU/g in RTE foods able to support the growth of *L. monocytogenes*, other than those intended for infants and for special medical purposes, when products are placed on the market or an absence in 25 g before the food has left the immediate control of the manufacturer. In this experiment, 100 CFU/g was chosen as a cut off as it would be unlikely for food to leave a processing plant at the starting levels of contamination used in this experiment, and this level would probably only occur after days of growth. The Food Safety Authority of Ireland (FSAI) “Survey on verification of compliance with Commission Regulation (EC) No 2073/2005 (12NS1)” in 2014 discussed the issue of categorisation of foods in terms of which EU criteria should apply. In the FSAI survey, coleslaw fell into food category 1.2, but if it had a shorter shelf-life (<5 days), it would have fallen into food category 1.3 and, therefore, *L. monocytogenes* would have been permitted at 100 CFU/g instead of a complete absence in 25 g. The average *L. monocytogenes* contamination of foods varies greatly and it has been found in foods from 10^2^ to >10^6^ CFU/g [19].

The difference in results of the checkerboard assays in broth and coleslaw could be due to temperature and pH. The concept of hurdle technology, the combination of a number of methods of preservation, is often used in food safety [20]. Alone, each hurdle may not be effective, but in combination, they can reduce bacterial growth. These hurdles can include temperature, pH, salt concentration, water activity, and preservatives. The pH of coleslaw is 3.9–4.5 [21,22], while the pH of TSB is 7.3. The TSB checkerboard was carried out at 30 °C compared to the coleslaw checkerboard, which was carried out at 4 °C. The low storage temperature and pH of coleslaw aid in reducing bacterial growth in the product. The temperature used for the experiment and the lower pH of the coleslaw may have reduced the MICs of P100 and Nisaplin^®^ alone and in combination in coleslaw compared to TSB. Also, it has been seen that nisin has increased activity against *L. innocua* at pH 5 compared to pH 7, which could have contributed to the difference between the checkerboard assays in broth and coleslaw [23]. The differences in results between food trials could be due to the effect of different foods and conditions on P100 activity. P100 had a significant effect on the growth of *L. monocytogenes* in melon and pear slices, but not on apple slices [24]. The same effect was seen in juices, in that P100 had a significant effect on the growth of *L. monocytogenes* in melon and pear juice but not in apple juice. Phage titre decreased in apple juice but stayed constant in melon and pear juice. The pH of apple slices and juice were determined to be 3.76 and 3.70, respectively, which is outside the range recommended by EFSA of pH 5.5–9.5 and an optimum of 7.7 [25]. The MICs of P100 and Nisaplin^®^ indicated by the checkerboard assay using TSB or coleslaw differed greatly. The checkerboard in broth indicated a much higher MIC for P100 and Nisaplin^®^ against ScottA and suggests that no synergistic killing effect would occur between them, while the same analysis using coleslaw indicated MICs 10 times lower for P100 and 8 times lower for Nisaplin^®^, with a synergistic effect. This could have been due to the increased growth of ScottA in broth, as it was carried out at a more optimum temperature for Listeria growth or that the growth media itself is optimised for rapid bacterial growth.

The liquid or solid nature of food can affect the diffusion of phages [26]. Phages can become bound to components of the food, and therefore unable to bind to the bacterial target. The volume of liquid present can also be an issue as, if too low a volume is present, phage will not be able to diffuse, but too great a volume can represent too large of a barrier for phage to cross. Also a low concentration of bacteria in food may mean that bacteria and phage may never meet by diffusion if phage concentration is low. It has been estimated that it would take in the order of 1000 years for 1 phage and 1 bacterium to meet within 1 mL of liquid. Therefore, in general, a high concentration of phage must be used to ensure a high likelihood that phage encounter the target bacterium. A lower concentration of phage may be required if bacterial numbers are high, as seen in our study.

It was not clear if phage killing over a period of 10 days was concentration-dependent. P100 at an MOI of 50 (*p* ≤ 0.001) or 2.5 (*p* ≤ 0.001) significantly and similarly reduced ScottA numbers in coleslaw over a 10-day period at 4 °C, a 5.85 log reduction and a 5.97, respectively. In comparing the reduction in ScottA between Day 0 and Day 1, where the greatest killing effect occurred, there was little difference in the killing between an MOI of 50 and an MOI of 2.5 (5.85 log reduction and 5.96 log reduction, respectively). A concentration-dependent killing of *L. monocytogenes* by P100 has been observed previously. In catfish fillets, P100 at a concentration of 2 × 10^3^ PFU/g did not reduce *L. monocytogenes* numbers inoculated at ~4.3log10 CFU/g, but 2 × 10^5^ PFU/g and 2 × 10^7^ PFU/g both worked, with 2 × 10^7^ PFU/g showing even greater reduction [27]. This was also seen in tuna slices, where P100 at a concentration of 5 × 10^8^ PFU/mL was better at reducing *L. monocytogenes* numbers inoculated at 100 CFU/g than P100 at 3 °C at a concentration of 10^5^ PFU/mL, representing MOIs of 10^6^ and 10^3^, respectively [28]. This trend was repeated in tuna slices at 3 °C inoculated with 6 log/g *L. monocytogenes* and treated with 5 × 10^8^ PFU/mL and 10^5^ PFU/mL, representing MOIs of 100 and 0.1, respectively, but the differences were not as pronounced. In some cases, P100 did not have a significant effect on *L. monocytogenes* at the end point of the trial, but was significant at points throughout the trial.

The reduction in ScottA was time-dependent; in both cases ScottA did not reach the EU regulation for *L. monocytogenes* in RTE foods until Day 10 of the trial. It should be borne in mind that we used an initial contamination level far in excess of anything that would be likely to occur in normal food production, and so we would expect excellent control with lower initial contaminating levels. As previously mentioned, in all cases, the greatest reduction in ScottA occurred in the first day after phage addition with a 5.85 log reduction with an MOI of 50, 5.96 log reduction with an MOI of 2.5 alone, and 5.97 log reduction 9.3 × 10^5^ CFU/g with an MOI of 2.5 in combination with Nisaplin^®^. After this point, the reduction was at a similar rate to that of negative controls. A similar rapid and dramatic reduction has previously been seen in trials using P100 in cabbage, smoked salmon, seafood, and hotdogs [29]. *L. monocytogenes* decreased from 1 × 10^3^ CFU/g by up to 1000-fold in hot dogs, 100-fold in mixed seafood and cabbage, and 50-fold in smoked salmon between Day 0 and 1, and this level of reduction was not seen again in each food during the 6-day trial. A rapid 10-fold reduction from 10^4^ CFU/g of *L. monocytogenes* after the addition of P100 in combination with *Lactobacillus sakei* was also seen in cooked ham [30]. When looking at the effect of phage contact time on bacterial reduction, Soni, Nannapaneni, and Hagens found that a contact time of 30, 60, or 120 min was more effective than 15 min contact time in reducing bacterial load on catfish fillets [27]. However, contact times of 30, 60, or 120 min were no more effective than one another. The titre of P100 at an initial MOI of 50 and MOI of 2.5 was stable throughout the experiment. The titre of commercially available Listex is 2 × 10^11^ PFU/mL and it can be added to food at a concentration of 1 × 10^9^ PFU/g, although it should be added with the estimated reduction in mind [25]. P100 could have been added at a higher titre to investigate if reduction would have occurred faster and reached the recognised EU food safety cut off earlier in the shelf life of the product.

In this experiment, Nisaplin^®^ alone did not significantly reduce ScottA numbers over the 10-day trial (*p* > 0.05). A significant reduction was not expected, as Nisaplin^®^ alone was added at sub MIC levels as established by a checkerboard assay in coleslaw and, of course, once again, we must note the extraordinarily high initial contamination levels. A combination of P100 and Nisaplin^®^ was significantly better than Nisaplin^®^ alone (*p* ≤ 0.05). Leverentz et al. found Nisaplin^®^ to be effective at reducing *L. monocytogenes* in sliced melon and apple pieces, but P100 in combination with Nisaplin^®^ was more effective [31]. Figueiredo and Almeida found that nisin and P100 individually inhibited growth of *L. monocytogenes* in RTE sliced ham [32]. A combination of P100 and nisin was significantly better at inhibiting growth of *L. monocytogenes* than nisin alone, but not P100 alone. In the trial carried out here, P100 and Nisaplin^®^ in combination were no more effective at reducing ScottA than P100 alone (*p* > 0.05).

The EFSA Panel on Biological Hazards [25] mentions the conditions of use of P100 to be between 1 and 35 °C, with an optimum temperature of 30 °C. P100 would not form plaques on *L. monocytogenes* 33116 ATCC 19117 at 37 °C, but would at 30 °C. This is consistent with the study of Tokman et al. [33]. The importance of the active temperature range of a phage should not be overlooked when developing phages for food safety, as phages may be required for use at refrigeration temperatures, at room temperature (in processing plants and in non-refrigerated foods), and at higher temperatures for lab assays.

A potential consequence of using phage in biopreservation and phage therapy is the possible emergence of phage resistant mutants [34]. This can be addressed in a number of ways. Firstly, bacterial mutants can often lose their phage-resistant phenotype when the phage is removed [35], because the evolution of phage resistance can reduce bacterial fitness or virulence [36]. Phages also have the ability to evolve past bacterial resistance mechanisms [37]. Methods to limit the emergence or reduce the impact of phage-resistant bacteria include the use of phage cocktails [38], the sequential use of different phages [29], the use of phage immediately before packaging to avoid the reintroduction of contamination that may have become phage resistant, and thorough cleaning of equipment to avoid phage resistant mutants forming and being introduced to food [39]. Phage-treated products should not be entered back into the production line, such as in “old–young smearing”, where mature cheeses are used to inoculate cheese that is in production [40]. Phage can also be used in combination with other compounds, such as essential oils, to avoid bacterial resistance [41].

The advantage of using a combination of antimicrobial compounds, or even a phage cocktail, can be seen from results of the rate of resistance to P100 assay and the coleslaw trial using P100 and Nisaplin^®^ in combination. Using an initial MOI of 50 or 2.5, no colonies resistant to P100 at an MOI of 50 were found after 8 or 10 days. The efficiency of plaquing of colonies from Day 8 and Day 10 were similar, with no major increase or decrease in efficiency of plaquing occurring between Day 8 and Day 10. However, colonies from the rate of resistance to P100 assay were found to have reduced sensitivity to P100—highlighting the fact that resistance can occur to P100, albeit only rarely. *L. monocytogenes* mutants resistant to 50 µg/mL nisin were previously found at a frequency of 10^−6^ to 10^−8^ [42]. Based on these prospective mutation rates to P100 and Nisaplin^®^, the chance of a strain developing resistance to both P100 and Nisaplin^®^ is extremely low. Therefore, the advantage of a combination lies in that any cells resistant to one antimicrobial that is present will be inhibited by the other and vice-versa.

## 5. Conclusions

Three objectives were outlined at the start of this study. They were (1) to investigate the effect of P100 alone at a high MOI in coleslaw against a pathogen of interest, (2) to find a suitable combination of concentrations of P100 and Nisaplin^®^ to act against the pathogen of interest in coleslaw, and (3) to investigate the efficacy of the combination in comparison to an untreated control, P100 alone and Nisaplin^®^ alone. P100 at an MOI of 50 significantly reduced *L. monocytogenes* numbers (*p* ≤ 0.001) in heavily contaminated coleslaw over a 10-day period and reached EU safe limits for *L. monocytogenes* in RTE-foods. A checkerboard assay was carried out and a combination of P100 at an MOI of 2.5 and 25 µg/mL Nisaplin^®^ was selected as a combination against *L. monocytogenes* in coleslaw. This combination was then used in a food trial against *L. monocytogenes*. P100 alone significantly reduced *L. monocytogenes* numbers (*p* ≤ 0.001), as did P100 and Nisaplin^®^ in combination (*p* ≤ 0.001). Nisaplin^®^ alone had no significant effect on *L. monocytogenes* numbers. P100 and Nisaplin^®^ in combination was more effective than Nisaplin^®^ alone, but not P100 alone. No resistance to P100 or Nisaplin^®^ was encountered at any point.

## Figures and Tables

**Table 1:** Listeria strains used and their sensitivity to phage P100. Efficiency of plaquing is represented as a fraction with standard error of the mean (SEM) of three separate experiments. If no P100 plaques formed on a strain, efficiency of plaquing is represented by (-).

Strain	Equivalent Names	Origin	Serotype	Efficiency of Plaquing of P100
*L. monocytogenes* ATCC 35152		Guinea pig [15]	1/2a	1.22 ± 0.4
*L. monocytogenes* 33116	ATCC 19117	Animal [16]	4d	1 ± 0
*L. monocytogenes* 33120	ATCC 19118	Animal [16]	4e	0.92 ± 0.54
*L. innocua* DPC 3372			-	0.83 ± 0.25
*L. monocytogenes* 33176	20240-954	Animal [16]	1/2b	0.72 ± 0.31
*L. monocytogenes* 33104	F2365, JI-119, TS43	California outbreak, 1985 [16]	4b	0.70 ± 0.03
*L. monocytogenes* ScottA	33013	Clinical (Massachusetts outbreak, 1983) [16]	4b	0.70 ± 0.03
*L. monocytogenes* EGDE		Rabbit [17]	1/2a	0.61 ± 0.2
*L. monocytogenes* 33007	RM2218	Food [16]	4b	0.59 ± 0.22
*L. monocytogenes* 33186	20674-01	Animal [16]	1/2b	0.49 ± 0.25
*L. monocytogenes* ATCC 15313		Rabbit [18]	1/2c	0.29 ± 0.02
*L. monocytogenes* ATCC 19112		Human CSF [15]	1/2c	0.24 ± 0.06
*L. monocytogenes* 6179		Cheese; production environment [17]	1/2a	0.002 ± 0.001
*L. grayi* CD671	ATCC 25400	Corn stalks [16]	-	-
*L. monocytogenes* 33028	OB001102	Food [16]	1/2b	-
*L. innocua* FA2039			-	-

**Table 2:** ScottA CFU/mL during P100 and Nisaplin^®^ checkerboard assay in coleslaw. MIC of P100 alone and Nisaplin^®^ alone are shown in bold. FIC values were calculated. FIC index results are interpreted as follows: FIC ≤ 0.5 is synergy, 0.5 < FIC ≤ 0.75 is partial synergy, 0.75 < FIC ≤ 1.0 is additive, FIC > 1.0 is indifferent, and FIC > 4 is antagonistic. The partially synergistic combination of P100 and Nisaplin^®^ is shown in red.

Antimicrobial Concentration	P100	P100	P100	P100	P100
	MOI 0	MOI 0.01	MOI 0.1	MOI 1	MOI 10
Nisaplin^®^ 0 µg/mL	9.6 × 10^5^	1.2 × 10^6^	1.1 × 10^6^	1.3 × 10^5^	**<1.0 × 10^4^** **MIC P100 Alone**
Nisaplin^®^ 12.5 µg/mL	5.0 × 10^5^	4.6 × 10^5^	6.8 × 10^5^	6.2 × 10^4^	<1.0 × 10^4^ FIC 1.25 Indifferent
Nisaplin^®^ 25 µg/mL	4.2 × 10^4^	8.0 × 10^4^	2.0 × 10^4^	**<1.0 × 10^4^** **FIC 0.6 Partial synergy**	<1.0 × 10^4^ FIC 1.5 Indifferent
Nisaplin^®^ 50 µg/mL	**<1.0 × 10^4^** **MIC Nisaplin^®^ Alone**	<1.0 × 10^4^ FIC 1.001 Indifferent	<1.0 × 10^4^ FIC 1.01 Indifferent	<1.0 × 10^4^ FIC 1.1 Indifferent	<1.0 × 10^4^ FIC 2 Indifferent
Nisaplin^®^ 100 µg/mL	<1.0 × 10^4^ FIC 2 Indifferent	<1.0 × 10^4^ FIC 2.001 Indifferent	<1.0 × 10^4^ FIC 2.01 Indifferent	<1.0 × 10^4^ FIC 2.1 Indifferent	<1.0 × 10^4^ FIC 3 Indifferent

## Supplementary Materials

The following are available online at https://www.mdpi.com/1999-4915/11/11/977/s1: Supplementary Table S1. Effect of P100 on ScottA in coleslaw food trial stored at 4 °C over a 10-day period; Supplementary Table S2. Efficiency of plaquing of 15 colonies picked from Day 8 and 15 colonies picked from Day 10 of P100 at an MOI of 50 treated coleslaw food trial to check for resistance to P100; Supplementary Table S3. Efficiency of plaquing of colonies isolated from phage seeded plates in the rate of resistance to P100 assay; Supplementary Table S4. Effect of P100 and Nisaplin^®^ in combination against ScottA in coleslaw food trial stored at 4 °C over a 10-day period; Supplementary Table S5. Efficiency of plaquing and Nisaplin^®^ sensitivity of colonies picked from Day 10 of combination food trail of Nisaplin^®^ alone, P100 alone, and P100 and Nisaplin^®^ in combination.
