# Supplementary material for: The Effect of a Commercially Available Bacteriophage and Bacteriocin on Listeria monocytogenes in Coleslaw
Source: Viruses. 2019 Oct 23;11(11):977. doi: 10.3390/v11110977 (PMC6893746; doi:10.3390/v11110977)
Supplement: Supplementary file 1 [file viruses-11-00977-s001.pdf]

viruses

*Article*

# Combining a commercially available bacteriophage and bacteriocin successfully reduces Listeria monocytogenes in coleslaw.

**Rhea Lewis [1,2], Andrei Sorin Bolocan[1,2], Lorraine A. Draper [1,2], R. Paul Ross[1,2] and Colin Hill [1,2]***

[1] APC Microbiome Ireland, University College Cork, Cork T12 YT20, Ireland
[2] School of Microbiology, University College Cork, Cork T12 YN60, Ireland
* Correspondence: c.hill@ucc.ie

*Supplementary tables and figures*

Supplementary Table S1. Effect of P100 on ScottA in coleslaw food trial stored at 4˚C over a 10 day period. P100 titre was also measured throughout the experiment.

| **Day** | **CFU/g** | |
|---|---|---|
| | **ScottA no P100** | **ScottA with phage P100** |
| 0 | 7.10E+05 | 7.10E+05 |
| 0.16 | 7.10E+05 | 2.00E+04 |
| 1 | 5.70E+05 | 1.70E+03 |
| 2 | 4.60E+05 | 1.20E+03 |
| 3 | 3.50E+05 | 8.30E+02 |
| 4 | 3.10E+05 | 3.70E+02 |
| 5 | 2.50E+05 | 4.00E+02 |
| 6 | 2.30E+05 | 1.50E+02 |
| 8 | 1.20E+05 | 2.90E+02 |
| 10 | 7.60E+04 | 0.00E+00 |

| **Day** | **PFU/g** |
|---|---|
| | **P100** |
| 0.16 | 3.80E+07 |
| 1 | 4.00E+07 |
| 2 | 3.00E+07 |
| 3 | 3.60E+07 |
| 4 | 3.40E+07 |
| 5 | 3.10E+07 |
| 6 | 2.10E+07 |
| 8 | 2.70E+07 |
| 10 | 1.60E+07 |

Supplementary Table S2. Efficiency of plaquing of 15 colonies picked from Day 8 and 15 colonies picked from Day 10 of P100 at an MOI of 50 treated coleslaw food trial to check for resistance to P100. Efficiency of plaquing is represented as a fraction with SEM of 3 separate experiments.

| Colonies picked from P100 at an MOI of 50 food trial | Efficiency of plaquing |
|---|---|
| Untreated Scott A | 1±0 |
| Day 8_1 | 0.81±0.1 |
| Day 8_2 | 0.67±0.1 |
| Day 8_3 | 0.89±0.11 |
| Day 8_4 | 0.71±0.04 |
| Day 8_5 | 0.95±0.15 |
| Day 8_6 | 0.84±0.17 |
| Day 8_7 | 0.84±0.17 |
| Day 8_8 | 0.89±0.11 |
| Day 8_9 | 0.75±0.05 |
| Day 8_10 | 0.89±0.11 |
| Day 8_11 | 0.92±0.21 |
| Day 8_12 | 0.83±0.26 |
| Day 8_13 | 1±0.19 |
| Day 8_14 | 1.03±0.17 |
| Day 8_15 | 0.95±0.15 |
| Day 10_1 | 0.95±0.15 |
| Day 10_2 | 1±0.19 |
| Day 10_3 | 0.95±0.15 |
| Day 10_4 | 0.92±0.21 |
| Day 10_5 | 1±0.19 |
| Day 10_6 | 1±0.19 |
| Day 10_7 | 1.25±0.14 |
| Day 10_8 | 1.14±0.25 |
| Day 10_9 | 1.14±0.25 |
| Day 10_10 | 1.03±0.18 |
| Day 10_11 | 1.08±0.21 |
| Day 10_12 | 1.08±0.21 |
| Day 10_13 | 1.14±0.25 |
| Day 10_14 | 1.33±0.17 |
| Day 10_15 | 1.28±0.31 |
| Day 10_16 | 1.17±0.25 |
| Day 10_17 | 1.22±0.28 |
| Day 10_18 | 1.28±0.31 |
| Day 10_19 | 1.17±0.25 |
| Day 10_20 | 1.14±0.25 |

Supplementary Table S3. Efficiency of plaquing of colonies isolated from phage seeded plates in the rate of resistance to P100 assay. Efficiency of plaquing is represented as a fraction. If no P100 plaques formed on a colony isolated from the rate of resistance to P100 assay efficiency of plaquing is represented by (-).

| **Colonies isolated from efficiency of lysogeny plate** | **Efficiency of plaquing** |
|---|---|
| Untreated ScottA | 1 |
| 1 | 0.26 |
| 2 | 1.38 |
| 3 | - |
| 4 | 0.0003 |
| 5 | 0.000388 |
| 6 | - |
| 7 | 0.000313 |
| 8 | - |
| 9 | - |
| 10 | - |
| 11 | 4.88 |
| 12 | - |
| 13 | - |
| 14 | - |
| 15 | 0.000463 |
| 16 | - |
| 17 | - |
| 18 | 5.38 |
| 19 | - |
| 20 | 0.0005 |

Supplementary Table S4. Effect of P100 and Nisaplin® in combination against ScottA in coleslaw food trial stored at 4°C over a 10 day period. P100 titre was also measured throughout the experiment.

| **Day** | **CFU/g** | | | |
|---|---|---|---|---|
| | **No P100 No Nisaplin®** | **P100 only** | **Nisaplin® only** | **P100 and Nisaplin®** |
| 0 | 9.40E+05 | 9.40E+05 | 9.40E+05 | 9.40E+05 |
| 0.16 | 9.40E+05 | 2.50E+05 | 2.00E+05 | 1.00E+05 |
| 1 | 5.10E+05 | 2.70E+04 | 7.40E+04 | 8.20E+03 |
| 2 | 4.60E+05 | 1.20E+04 | 5.70E+04 | 4.40E+03 |
| 3 | 3.50E+05 | 5.90E+03 | 5.50E+04 | 3.10E+03 |
| 4 | 2.80E+05 | 3.70E+03 | 4.70E+04 | 1.60E+03 |
| 5 | 2.40E+05 | 2.20E+03 | 3.20E+04 | 8.00E+02 |
| 6 | 2.00E+05 | 1.50E+03 | 1.80E+04 | 4.80E+02 |
| 8 | 1.20E+05 | 3.60E+02 | 2.80E+04 | 2.60E+02 |
| 10 | 7.40E+04 | 2.10E+02 | 1.40E+04 | 1.20E+02 |

| **Day** | **PFU/g** | |
|---|---|---|
| | **P100 only** | **P100 and Nisaplin®** |
| 0.16 | 2.70E+06 | 2.50E+06 |
| 1 | 3.10E+06 | 3.70E+06 |
| 2 | 3.20E+06 | 3.70E+06 |
| 3 | 4.00E+06 | 4.70E+06 |
| 4 | 3.70E+06 | 3.70E+06 |
| 5 | 3.10E+06 | 3.50E+06 |
| 6 | 2.60E+06 | 3.10E+06 |
| 8 | 2.70E+06 | 2.50E+06 |
| 10 | 3.10E+06 | 2.60E+06 |

Supplementary Table S5. Efficiency of plaquing and Nisaplin® sensitivity of colonies picked from Day 10 of combination food trail of Nisaplin alone, P100 alone and P100 and Nisaplin® in combination. Efficiency of plaquing is represented as a percentage with SEM of 3 separate experiments.

| Colonies picked from Day 10 of combination food trial | Efficiency of plaquing | Nisaplin® zone of inhibition diameter (mm) |
|---|---|---|
| Untreated ScottA | 1±0 | 10.06 |
| P100 alone_1 | 0.90±0.05 | 10.04 |
| P100 alone_2 | 0.89±0.06 | 10.15 |
| P100 alone_3 | 1±0 | 9.9 |
| P100 alone_4 | 0.93±0.07 | 10.56 |
| P100 alone_5 | 0.98±0.1 | 9.47 |
| P100 alone_6 | 0.98±0.1 | 10.08 |
| P100 alone_7 | 0.93±0.07 | 9.75 |
| P100 alone_8 | 1±0 | 9.38 |
| P100 alone_9 | 1±0 | 10.15 |
| P100 alone_10 | 1±0 | 9.86 |
| Nisaplin® alone_1 | 1±0 | 10.45 |
| Nisaplin® alone_2 | 1±0 | 10.49 |
| Nisaplin® alone_3 | 0.95±0.05 | 10.45 |
| Nisaplin® alone_4 | 1±0 | 10.4 |
| Nisaplin® alone_5 | 0.78±0.06 | 10.52 |
| Nisaplin® alone_6 | 0.93±0.07 | 9.71 |
| Nisaplin® alone_7 | 0.94±0.06 | 10.37 |
| Nisaplin® alone_8 | 1±0 | 10.39 |
| Nisaplin® alone_9 | 0.93±0.07 | 9.77 |
| Nisaplin® alone_10 | 1.05±0.05 | 10.07 |
| P100 and Nisaplin®_1 | 0.95±0.05 | 9.55 |
| P100 and Nisaplin®_2 | 0.83±0.02 | 10.09 |
| P100 and Nisaplin®_3 | 0.95±0.05 | 10.09 |
| P100 and Nisaplin®_4 | 0.85±0.08 | 10.27 |
| P100 and Nisaplin®_5 | 0.90±0.05 | 9.57 |
| P100 and Nisaplin®_6 | 0.95±0.05 | 9.67 |
| P100 and Nisaplin®_7 | 0.95±0.05 | 9.7 |
| P100 and Nisaplin®_8 | 0.90±0.05 | 9.19 |
| P100 and Nisaplin®_9 | 0.89±0.06 | 9.79 |
| P100 and Nisaplin®_10 | 0.95±0.05 | 9.26 |

© 2019 by the authors. Submitted for possible open access publication under the terms and conditions of the Creative Commons Attribution (CC BY) license (http://creativecommons.org/licenses/by/4.0/).
